# Effect of Sn Addition on Microstructure and Mechanical Properties of Sintered Ti_2_AlNb-Based Alloys

**DOI:** 10.3390/ma18030715

**Published:** 2025-02-06

**Authors:** Zhu Li, Yaran Zhang, Xifeng Yan, Guoqing Xia, Qilin Yu, Xinze Li, Qi Cai

**Affiliations:** 1 School of Materials Science and Engineering, Beijing Institute of Technology, Beijing 100081, China; 3120231050@bit.edu.cn (Z.L.);; 2 Material Science Research Center, Xingtai University, Xingtai 054001, China; 3 Tianjin Key Laboratory of Integrated Design and On-line Monitoring for Light Industry & Food Machinery and Equipment, School of Mechanical Engineering, Tianjin University of Science and Technology, Tianjin 300457, China

**Keywords:** Ti_2_AlNb alloys, Sn addition, powder metallurgy, microstructure, mechanical properties, strengthening mechanisms

## Abstract

Using cold isostatic pressing and atmospheric pressure sintering, Ti-18Al-28Nb-xSn alloys were synthesized by incorporating 0.5 at.%, 1 at.%, 2 at.%, and 4 at.% Sn powder into Ti, Al, and Nb powders. This study investigated the effects of Sn concentration on the microstructure and mechanical properties of Ti_2_AlNb-based alloys, with a particular focus on the underlying strengthening mechanisms. X-ray diffraction (XRD) analysis identified α_2_, O, and B2 as the primary phases in the alloy and demonstrated that Sn addition significantly influenced the proportions of these phases, thus impacting the overall mechanical performance of Ti_2_AlNb-based alloys. The optimal combination of elasticity, strength, and plasticity was achieved at a Sn concentration of 1 at.%; at this time, the elastic modulus of the alloy was 26.8 GPa, with a compressive strength of up to 1352 MPa and a fracture strain of 42.8%. However, further increases in Sn content beyond this level led to reductions in both strength and plasticity. At Sn concentrations above 2 at.%, increased porosity and the formation of micropores were observed, facilitating microcrack aggregation and fracture, which ultimately compromised the alloy’s mechanical integrity. By exploring the intrinsic strengthening mechanisms, this study tries to understand the influence of Sn on the strengthening effects and to optimize the content range of Sn addition to ensure the best strengthening effect and good density are shown in high-Nb-content TiAl alloy, providing a reference for future research in this field.

## 1. Introduction

TiAl-based alloys, as a kind of structural materials, are extensively utilized in the aerospace and automotive industries owing to their low density, high specific strength, oxidation resistance, and superior high-temperature performance [1,2,3]. To enhance the strength and ductility of these alloys for broader industrial applications, heat treatment [4], rolling [5], directional solidification [6], and powder metallurgy [7] have been employed. Nevertheless, the mechanical strength of conventional TiAl-based alloys remains insufficient to meet stringent production requirements. Alloying is therefore widely adopted to improve TiAl alloy properties. TiAl alloys inherently contain certain amounts of β/B2 and α_2_-Ti_3_Al phases, with the β phase offering relatively high ductility at elevated temperatures, thus facilitating thermomechanical processing. The addition of β phase stabilizing elements such as Cr, V, Nb, Mo, and Mn enhances the malleability and mechanical properties of TiAl alloys [8,9]. In particular, third-generation TiAl alloys with high Nb contents exhibit superior yield strength, tensile strength, creep resistance, and oxidation resistance and elevated service temperatures compared to conventional TiAl alloys, making them a current research focus [4,10]. However, synthesizing high-Nb-content TiAl alloys presents challenges; traditional melting and casting techniques often lead to segregation and limited plasticity, hindering deformation processability [11]. Additionally, in high-Nb-content TiAl-based alloys, Nb promotes the segregation of alloying elements and increases β/B2 phase content within the matrix. While β/B2 phases enhance high-temperature processing, they detrimentally affect room temperature plasticity and high-temperature creep resistance and thus are undesirable at lower temperatures [12,13,14]. Due to the sensitivity of the microstructure of TiAl-based alloys to the addition of Nb content, it is often necessary to improve the plasticity and strength of high-Nb-content TiAl alloys through grain refinement and the addition of alloying elements.

Recently, powder metallurgy (PM) techniques have been employed to produce alloys with refined grain structures and chemically uniform microstructures, effectively addressing issues of segregation within the crystal lattice. However, this method is not directly applicable to synthesizing TiAl alloys, as the TiAl alloy indeed struggles to achieve high density through traditional compaction and sintering processes. The sintering properties of TiAl alloys must be enhanced through doping with transition metals, such as copper (Cu), cobalt (Co), or nickel (Ni), which are commonly used in alloy synthesis [15,16,17]. These transition metals significantly increase the density of pre-alloyed powders and readily form intermetallic compounds with Ti and Al. However, these compounds can be brittle or cause grain boundary segregation, ultimately reducing the alloy’s mechanical performance. Now, an alloying element that can replace transition metals to improve sintering density and enhance the overall mechanical properties of high-Nb-content TiAl-based alloys without segregation is required. Due to the low melting point of Sn, it can exist in a liquid phase during sintering, providing good densification enhancement and strengthening effects; therefore, it is regarded as a promising liquid phase sintering additive [18,19,20]. The addition of Sn effectively lowers the melting point of high-Nb-content TiAl-based alloys and enhances sintering behavior without affecting the mechanical properties of the alloy. Moreover, as a kind of neutral element, atoms of Sn can occupy the Al sublattice rather than that of Ti due to Al and Sn belong to the same group of post-transition metals, and the atomic radius of Sn is larger than that of Al, so Sn usually exhibits a significant solid solution strengthening effect in TiAl alloys, increasing their high-temperature strength and hardness [21,22,23,24]. In the various powder metallurgy methods, spark plasma sintering (SPS) and hot isostatic pressing (HIP) are the most common, but they are relatively expensive and their preparation processes are complicated [25,26]. Applying a certain pressure to the pre-alloyed powder in conjunction with pressureless sintering, in particular, represents the simplest and most cost-effective PM process for TiAl alloy preparation [15,27,28,29]. In addition, based on the principle of liquid phase sintering, the addition of Sn can also compensate for the density deficiency of pressureless sintering [30]. Pan et al. [31] observed that adding 1 at.% Sn powder as a strengthening sintering agent produced a uniform, fine α2/γ full lamellar structure in the alloy using pressureless sintering. The presence of Sn reduced the sintering densification temperature, improved the billet densification, decreased linear shrinkage, and facilitated grain size reduction. Consequently, Sn-doped alloys demonstrated increased hardness, compressive strength, yield strength, and compression ratio compared to those without Sn, with no apparent effects on fracture behavior. Thereby, the method of applying certain pressure to the pre-alloyed powder followed by pressureless sintering is a good approach to prepare TiAlNb-xSn.

Sn can enhance the overall mechanical properties of high-Nb-content TiAl-based alloys as an alloying element, and it can also serve as a sintering additive to improve sintering density, reducing porosity in alloys obtained through powder metallurgy. However, there are currently few studies on the composition of quaternary alloys formed by adding Sn to high-Nb-content TiAl-based alloys, and it remains unclear how variations in Sn content affect the microstructural evolution and mechanical properties of high-Nb-TiAl alloys obtained through powder sintering methods.

This study aims to systematically investigate the influence of different Sn contents (0.5 at.%, 1 at.%, 2 at.%, and 4 at.%) on the microstructural evolution and mechanical properties of high-Nb-content TiAl-based alloys using Ti-18Al-28Nb pre-alloyed powder as the raw material, with Sn serving both as a strengthening element and a sintering additive, utilizing cold isostatic pressing followed by pressureless sintering. By exploring the dual role of Sn in the quaternary alloy (Ti-Al-Nb-Sn), this research seeks to provide new insights for optimizing high-Nb-TiAl alloys. It aims to clarify the role of Sn in the microstructural evolution of the alloy, analyze the intrinsic strengthening mechanisms that lead to the final enhancement of the alloy, and provide quantitative data to understand the influence of Sn on the strengthening effects. This will help to optimize the range of Sn addition to ensure the best strengthening effect and provide a reference for future research in this field, facilitating a balance between performance and cost for the practical application of high-Nb-TiAl alloys.

## 2. Materials and Methods

In this experiment, titanium powder (~45 μm, purity > 99.90%, supplied by the Beijing Xing Rong Yuan Technology Co. Ltd., Beijing, China), aluminum powder (5–10 μm, purity > 99.90%, supplied by Shanghai Yunfu Nanotechnology Co. Ltd., Shanghai, China), niobium powder (5–10 μm, purity > 99.90%, supplied by Shanghai Yunfu Nanotechnology Co. Ltd.), and Sn powder (~45 μm, purity > 99.95%, supplied by the Beijing Xing Rong Yuan Technology Co. Ltd.) were used to prepare alloys following the stoichiometric ratio of Ti-18Al-28Nb-xSn (x = 0, 0.5, 1.0, 2.0, and 4.0 at.%). The Ti, Al, Nb, and Sn powders were loaded into ball milling jars and mixed with a ball-to-powder ratio of 5:1 in a planetary ball mill, operating for 1 h at a rotational speed of 180 r/min. The mixed powders were then placed into rubber molds, vacuum-sealed, and subjected to cold isostatic pressing at 400 MPa for 40 min. The resulting compacts were removed from the molds and placed in a vacuum sintering furnace, where argon was used as a protective atmosphere during sintering. The sintering process was conducted at 1400 °C with a 3 h hold time, after which the furnace was cooled to room temperature before sample removal. The prepared sample were named S1, S2, S3, S4, and S5, and their chemical compositions are shown in Table 1.

The phase compositions of the alloys were analyzed using X-ray diffraction (XRD, Bruker D8 Advance, Billerica, MA, USA), with phase volume fractions calculated according to the Rietveld method. After grinding, polishing, and etching, the samples were observed under an optical microscope (OM, ZEISS Axio Observer A1m, Oberkochen, Germany) and a scanning electron microscope (SEM, Gemini SEM 300) to examine the microstructure at low and high magnifications, respectively. The distribution of Sn in each phase was analyzed using an energy-dispersive spectrometer (EDS, AZtec X-Max 50, London, UK). Image-Pro Plus software (lmage-Pro Plus 6.0) was utilized to measure the porosity in optical micrographs and to determine the volume fraction and size of the α_2_ and O phases in SEM images. The mechanical properties of the alloys were evaluated using a universal electronic testing machine (Instron 5985, Norwood, MA, USA), and quasi-static compressive properties were measured on cylindrical specimens with a diameter and length of 5 mm.

## 3. Results and Discussion

### 3.1. Effect of Sn Content on Phase Composition of Alloy

**Figure 1:**
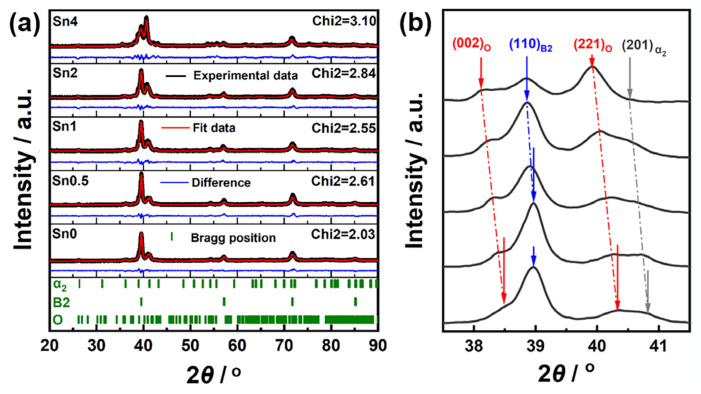
XRD patterns of the Ti-18Al-28Nb-xSn (x = 0, 0.5, 1.0, 2.0, and 4.0) alloys: (**a**) XRD patterns of the alloy with varying Sn; (**b**) the changes in the diffraction peak intensities of phases as a function of 2θ.

The XRD patterns of the alloy with varying Sn content are presented in Figure 1a, showing the presence of B2 (Ti, PDF.# 44-1288), α_2_ (Ti_3_Al, PDF.# 14-0451), and O (Ti_2_AlNb, PDF.# 49-1449) phases. Figure 1b illustrates the changes in the diffraction peak intensities of these phases as a function of 2θ. After Sn doping, the 2θ values corresponding to each phase’s diffraction peaks shifted slightly to the left, indicating an increase in the crystallographic interplane spacing.

**Figure 2:**
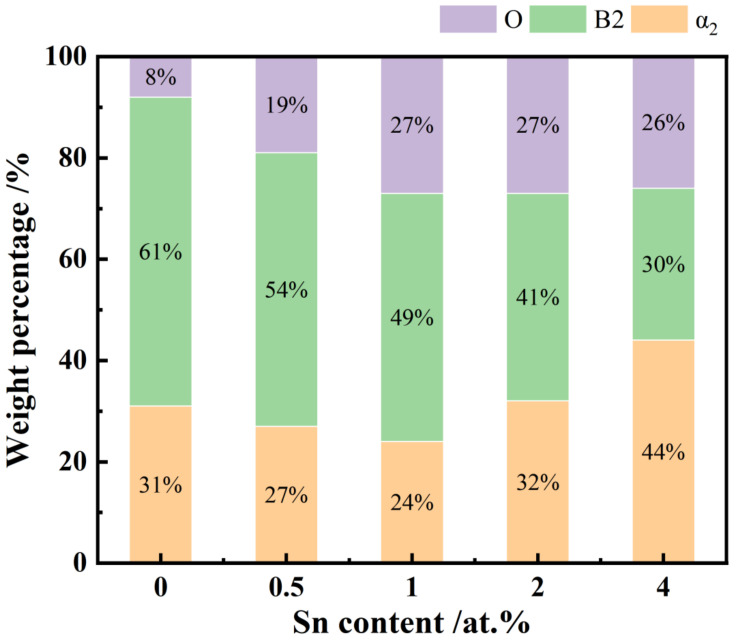
The phase compositions of the samples with various Sn dopings.

To obtain quantitative data on the phase composition, the volume fractions of each phase were calculated using the Rietveld method, as shown in Figure 2.

Combined analysis of Figure 1a and Figure 2 reveals that the sample without Sn addition predominantly comprises B2 and α_2_ phases, with minimal O phase presence, and the diffraction peak corresponding to the O phase’s (221) crystal plane appears only at 40.3°. As Sn content increases, the proportion of the O phase in the alloy also increases, while the proportions of the B2 and α_2_ phases decrease. Beyond 1 at.% Sn, the O phase fraction stabilizes, while the B2 phase continues to decrease and the α_2_ phase begins to increase; at 4 at.% Sn, the α_2_ phase proportion reaches its maximum.

These phase transformations can be attributed to the effects of Sn as a neutral element with high solubility in both the B2 and α_2_ phases. The atomic radius of Sn is larger than those of Ti and Al, and Sn doping causes lattice distortion in all phases, generating additional distortion energy. As shown in Figure 1b, the 2θ shift for B2 and α_2_ phases is less than that for the O phase, indicating greater distortion energy, which promotes the transformation of the B2 and α_2_ phases into the O phase. When Sn content is increased to 0.5 at.%, no change occurs in the peak position of the B2 phase’s (110) plane, suggesting that Sn initially affects the lattice of the α_2_ and O phases preferentially. The B2 phase’s peak position and interplanar spacing remain unchanged until Sn content reaches 1 at.%, suggesting that the transformation from the α_2_ to O phase occurs independently of B2. Consequently, the phase transition sequence at a low Sn content begins with α_2_ → O, followed by B2 → O or B2 + α_2_ → O. As the Sn content reaches 2 at.%, the O phase stabilizes, the B2 phase continues to decrease, and the α_2_ phase increases, indicating that Sn addition facilitates the B2 → α_2_ transformation.

### 3.2. Effect of Sn Content on Microstructure of Alloys

**Figure 3:**
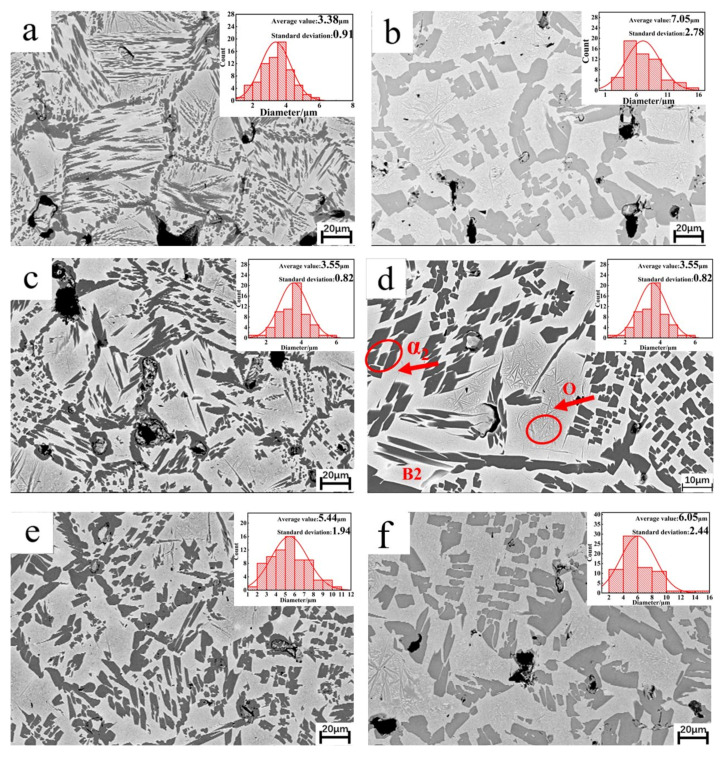
The SEM image of the samples (size distribution histogram of grain boundary α_2_ is shown in upper right corner of image): (**a**) S1; (**b**) S2; (**c**) S3; (**d**) magnified view of S3; (**e**) S4; (**f**) S5.

Low-magnification SEM images of the alloy are presented in Figure 3. The dark gray regions represent the α_2_ phase, the light gray matrix corresponds to the B2 phase, and the intermediate gray regions, which appear needle-like, represent the O phase. In the alloy without Sn addition, the B2 phase forms the matrix, with a small amount of fine, needle-like O phase distributed within it. A portion of the α_2_ phase is present in lamellar form within the grains, while a minor portion appears in equiaxed form, primarily located at the B2 phase grain boundaries, forming a network structure. The average size of the α_2_ phase in this alloy is 3.38 ± 0.91 μm. With the addition of 0.5 at.% Sn, the α_2_ phase at the grain boundaries becomes significantly coarser, reaching an average size of 7.05 ± 2.78 μm.

**Figure 4:**
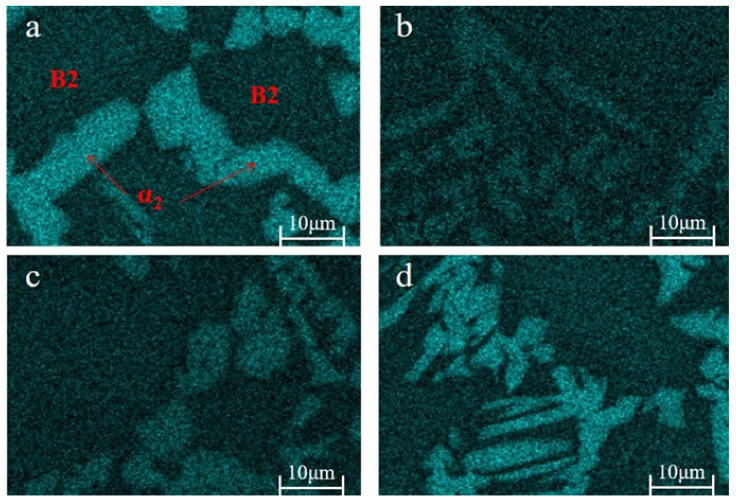
The Sn distribution in the samples’ microstructure: (**a**) S2; (**b**) S3; (**c**) S4; (**d**) S5.

Figure 4 shows the EDS mapping of Sn in the alloys, where the α_2_ regions appear brighter than the B2 regions, indicating more substantial Sn diffusion into the α_2_ phase compared to the B2 phase. The atomic percentages of each element in the α_2_ phase and O phase are shown in Table 2 and Table 3. The atomic percentage of Sn in the α_2_ phase is higher than the O phase and the whole samples. Moreover, the atomic ratios of each phase are generally consistent with the XRD results. Figure 4a reveals enhanced brightness at the grain boundaries, suggesting a higher Sn concentration in these areas, which promotes the growth of α_2_ at the boundaries. Conversely, Sn solubility in the coarse acicular and equiaxed α_2_ phases within the grains is limited. The higher concentration of defects and increased distortion energy at the grain boundaries provide a substantial driving force for atomic diffusion, facilitating α_2_ phase nucleation and growth prior to the B2 phase. Thus, Sn exerts a more pronounced effect on α_2_ phase size at the grain boundaries. Additionally, the content of the O phase increases at 0.5 at.% Sn. Comparing Figure 3a,b, the distribution of the O phase becomes more apparent, which agrees with the XRD analysis. This result confirms the occurrence of the α_2_ → O transformation. At 1 at.% Sn, the portions of the α_2_ phase at the grain boundaries and the lamellar α_2_ phase within the grains fracture, resulting in discontinuous equiaxed shapes with an average size of 3.55 ± 0.82 μm. At this stage, Sn begins to incorporate into the B2 phase, and the O phase content continues to rise. The increase in O phase content results from phase transformations, including B2 → O, B2 + α_2_ → O, and α_2_ → O. The B2 + α_2_ → O transition, a peritectoid reaction, often produces a peritectoid ring band of O phase around the α_2_ phase, known as the Rim O phase [32]. However, no such Rim O phase structure is observed in Figure 3c, suggesting that the intragranular transformation is likely B2 → O rather than B2 + α_2_ → O. As Sn content increases to 2 at.%, the fractured α_2_ phase at the grain boundaries grows to an average size of 5.44 ± 1.94 μm, reaching 6.05 ± 2.44 μm at 4 at.% Sn. At this stage, both grain boundary and intragranular α_2_ phases adopt an equiaxed, uniform distribution within the B2 matrix, with only minor remnants of the lamellar α_2_ phase. The O phase content stabilizes and ceases to increase, while the intragranular phase transformation is primarily B2 → α_2_. In summary, as Sn content increases, the proportion of the α_2_ phase initially decreases, then increases, ultimately adopting an equiaxed shape and a uniform distribution. Simultaneously, the proportion of the O phase initially increases and then stabilizes. The phase transformations include B2 → O, α_2_ → O, and B2 → α_2_.

**Figure 5:**
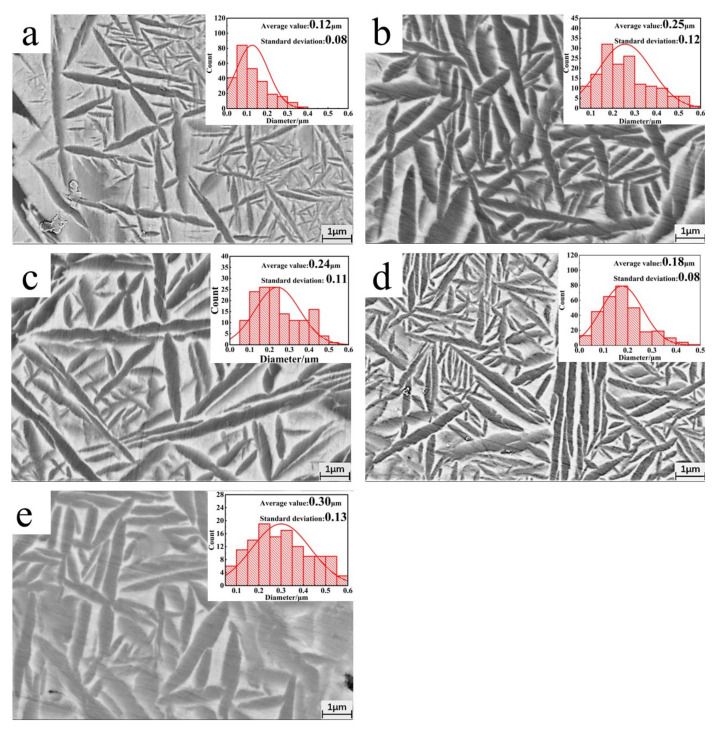
An SEM image of the O phase in the various samples (distribution histogram of the O phase size is shown in the upper right corner of the image): (**a**) S1; (**b**) S2; (**c**) S3; (**d**) S4; (**e**) S5.

**Figure 6:**
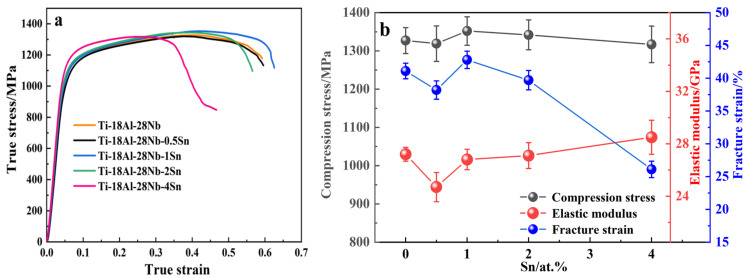
The compression properties of the samples: (**a**) the RT compression strain–-stress curve of the samples; (**b**) the compression properties of the samples.

Figure 5 presents high-magnification SEM images of the alloy microstructure, highlighting the acicular O phase within grains and the B2 phase matrix, which together form a Widmanstätten structure. As shown in the O phase size distribution diagram in Figure 6, the addition of Sn increases both the content and size of the O phase, which in turn enlarges the dimensions of the Widmanstätten structure. However, with further increases in Sn content, the O phase size reaches a plateau and no longer significantly affects the overall dimensions of the Widmanstätten structure.

### 3.3. Effect of Sn Content on Compressive Mechanical Properties of Alloys

Figure 6a presents the compressive true stress–strain curves for alloys with varying Sn contents. The addition of Sn at different concentrations does not result in a noticeable change in the alloy’s compressive strength. However, when the Sn content reaches 4 at.%, a slight increase in strength is observed. Simultaneously, the critical fracture strain decreases to a range of 0.45 to 0.5, leading to a reduction in plasticity.

As shown in Figure 6b, although the modulus of elasticity curve for the alloy exhibits slight fluctuations, the values generally remain within the range of 24.5 GPa to 28.5 GPa. In Figure 6a, it is evident that the elastic deformation regions of the compression curves for several samples overlap significantly, indicating similar slopes and minimal differences in the values of the modulus of elasticity (E). The relatively low and consistent modulus of elasticity of the alloy is primarily determined by the B2 phase, as this phase constitutes a significant portion of the matrix. The modulus of elasticity of the alloy is thus governed by that of the B2 phase, which, in comparison to other phases, has a lower modulus of elasticity [33].

**Figure 7:**
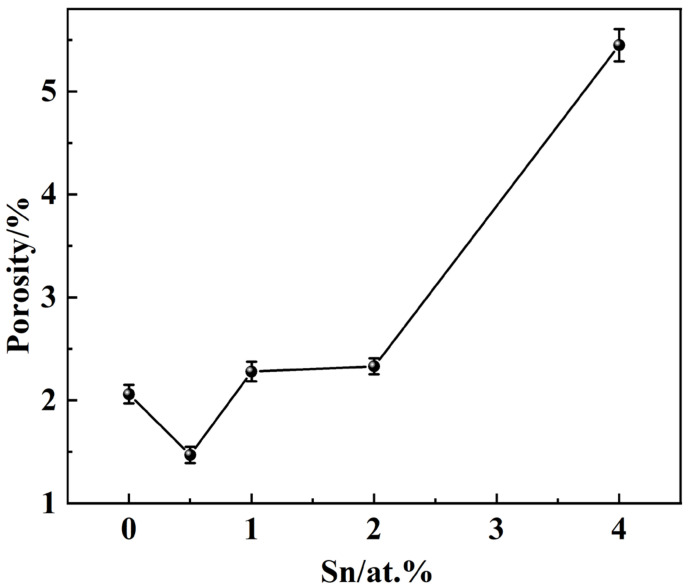
The porosity of the samples.

When the Sn content is low (0.5 at.%), both the compressive stress and critical fracture strain of S2 decrease. This is attributed to the growth of the α_2_ phase at the grain boundaries, which reduces the strength and plasticity of S2. Upon increasing the Sn content to 1 at.%, the reticular α_2_ precipitates at the grain boundaries fragment and decrease in size. Consequently, the compressive stress and critical fracture strain of S3 increase significantly compared to S2. At this point, the alloy exhibits a balance between elasticity, plasticity, and strength, resulting in optimal overall mechanical properties; at this time, the elastic modulus of the alloy is 26.8 GPa, with a compressive strength of up to 1352 MPa and a fracture strain of 42.8%. Further increasing Sn to 2 at.% leads to the discontinuity of the reticular α_2_ precipitates at the grain boundaries in S4, which gradually adopt an isometric shape. As the precipitates grow larger, the compressive stress of the alloy decreases, though it remains higher than the initial level. The critical fracture strain shows only a slight reduction compared to the Sn-free alloy, and the overall mechanical properties begin to deteriorate. When the Sn content reaches 4 at.%, both the elastic modulus and critical fracture strain values are lower than those of the original specimen, indicating a substantial decline in performance. The isometric α_2_ phase at the grain boundaries and within the grains of S5 is the largest among all samples, causing both the strength and plasticity of S5 to be terrible. As shown in Figure 7, the porosity decreases to 1.47% in S2, and the sintered density is the best. The porosities of S3 and S4 are not much different from that of S1, which has a porosity of 2.06%, indicating good density. The porosity of S5 is considerably increased up to 5.45%, and this factor also contributes to sharp declines in both the strength and plasticity of S5.

In summary, keeping the Sn content below 2 at.% can ensure both the mechanical properties and density of the alloy well without causing segregation, playing a role in enhancing the strengthening and aiding the sintering process. When the Sn content is 4 at.%, both the mechanical properties and density of the alloy are poor.

**Figure 8:**
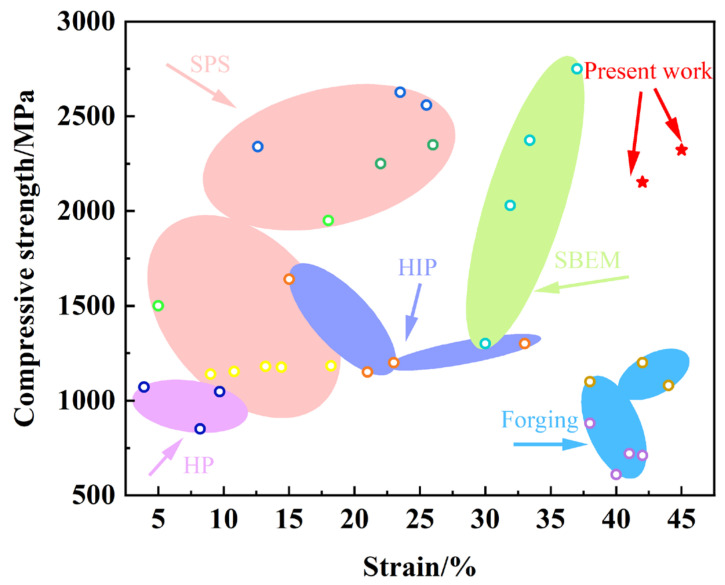
A comparison of the ultimate strength and the strain-at-fracture samples of this research and those reported in other references [34,35,36,37,38,39,40,41,42,43,44,45].

To further demonstrate the performance of high-Nb-content TiAl-based alloys doped with Sn elements prepared by the cold isostatic pressing combined with pressureless sintering process, we conducted a deep comparison with other common titanium alloy materials made from different compositions and processes [34,35,36,37,38,39,40,41,42,43,44,45]. The results are shown in Figure 8. In terms of processing, the alloys produced in this study exhibit significantly higher compressive strengths and superior plasticities than those formed by traditional forging, hot pressing sintering (HP), and hot isostatic pressing (HIP). Additionally, the mechanical properties of the alloys obtained through spark plasma sintering (SPS) and this study are quite similar; however, the cold isostatic pressing combined with pressureless sintering process is simpler and more cost-effective. From a compositional perspective, the Ti-18Al-28Nb-xSn alloy in this study displays superior mechanical properties and has the potential to replace some Ti-Al-V alloys, thus effectively addressing the potential toxicity issues associated with the addition of vanadium (V) [46,47]. This advancement provides an opportunity for the application of this alloy in the biomedical field, promoting the research and development of new materials with theoretical significance and practical prospects.

**Figure 9:**
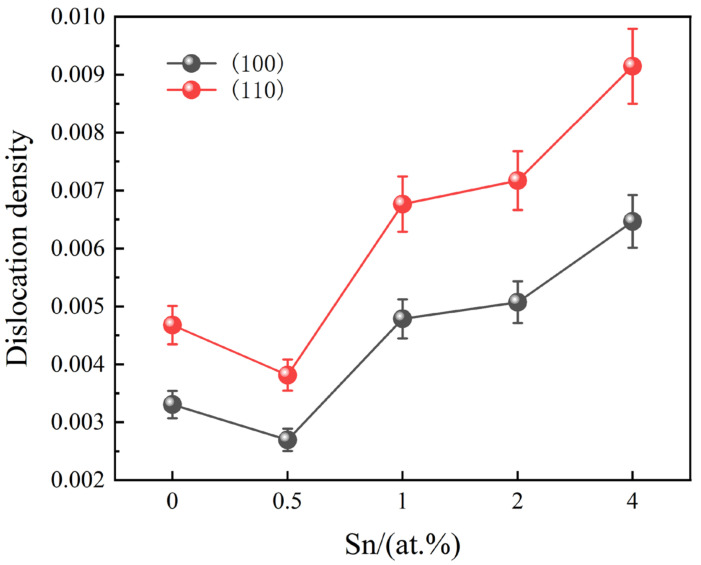
The dislocation density in the B2 phase with different Sn contents.

To investigate the underlying reasons for the changes in the mechanical behavior of the alloys after Sn addition, the strengthening mechanism of the alloys is examined. In all specimens, the B2 phase content is notably high, and the dislocation state within this phase plays a crucial role in determining the alloy’s strength. There is a direct correlation between dislocation density and alloy strength: as the dislocation density increases, the strength of the alloy also increases. Consequently, the dislocation densities ρ of the main crystallographic planes (100) and (110) within the B2 phase were calculated, and the results are presented in Figure 9.

Based on the calculated dislocation density of the B2 phase, as well as the dimensions of the O phase and the grain boundary α_2_ phase, the strengthening mechanisms of the alloy and their contributions were determined.

**Figure 10:**
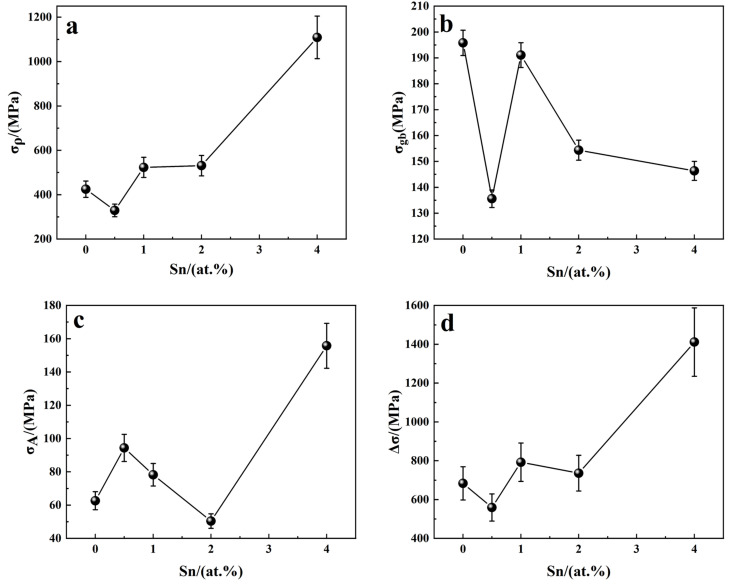
Relationship between Sn content and strengthening mechanism: (**a**) σ_ρ_-Sn; (**b**) σ_gb_-Sn; (**c**) σ_A_-Sn; (**d**) Δσ-Sn.

The results of these calculations, shown in Figure 10, reveal that several strengthening mechanisms undergo notable changes at S2 with the addition of 0.5 at.% Sn. The calculated results are consistent with the findings from quasi-static compression experiments, confirming the accuracy of the calculations. At 0.5 at.% Sn, the dislocation density within the B2 matrix phase decreases, weakening the dislocation strengthening effect (σ_ρ_). Additionally, the size of the grain boundary α_2_ phase increases, reducing the grain boundary strengthening effect (σ_gb_). Although the fine needle-like O phase can pin dislocation motion, its weight percentage is only 8%, which is insufficient to exert a dominant influence. While the strengthening effect of the O phase (σ_A_) increases, the overall strength and plasticity of S2 decline compared to S1.

When the Sn content increases to 1 at.%, the dislocation density in the B2 phase continues to rise, enhancing σ_ρ_, while the α_2_ phase at the grain boundaries forms a network structure. Due to the fragmentation of α_2_, σ_gb_ begins to increase. The O phase content rises to 19%, but σ_A_ still does not serve as the primary strengthening mechanism. At this stage, the strength and plasticity of S3 begin to improve. As the Sn content continues to increase, the dislocation strengthening effect for S4 reaches a plateau, and the main strengthening mechanisms, σ_gb_ and σ_A_, show a downward trend. Although the combined effect of the strengthening mechanisms in S5 is relatively favorable, the porosity in S5 is much higher than in the other samples. These micropores tend to aggregate, leading to the formation of microcracks and eventual fracture. Consequently, the mechanical properties of both S4 and S5 are significantly degraded.

## 4. Conclusions

This study offers the following conclusions:

1. The phase compositions of Ti-18Al-28Nb-xSn (x = 0, 0.5, 1.0, 2.0, and 4.0 at.%) alloys, fabricated through the powder metallurgical process of “cold isostatic pressing + atmospheric pressure sintering”, consist of α_2_, O, and B2 phases. As the Sn content increases, the proportion of the α_2_ phase decreases initially and then increases, ultimately adopting an isometric shape with a diffuse distribution. The proportion of the O phase increases before stabilizing, while the B2 phase content consistently decreases. The phase transformation sequence is B2 → O, α_2_ → O, and B2 → α_2_.
2. The microstructure of the Ti-18Al-28Nb alloy without Sn addition exhibits a Widmanstätten structure composed of B2 + O phases, with an equiaxed α_2_ phase. The acicular O phase is diffusely distributed within the B2 matrix. The addition of Sn promotes the growth of the α_2_ phase at the grain boundaries, leading to an increase in the sizes of both the α_2_ and O phases at these boundaries.
3. The mechanical properties of the alloys are significantly enhanced with the addition of Sn. When the Sn content reaches 1 at.%, the alloy exhibits optimal overall mechanical properties, characterized by a favorable balance of elasticity, strength, and plasticity; at this time, the elastic modulus of the alloy is 26.8 GPa, with a compressive strength of up to 1352 MPa and a fracture strain of 42.8%. Further Sn addition results in reductions in strength and plasticity, although elasticity improves. Additionally, when Sn content exceeds 2 at.%, the alloy’s porosity increases substantially, leading to the formation of numerous micropores. These micropores are prone to coalescing into microcracks, which ultimately causes fracture, thereby compromising the alloy’s strength and plasticity and adversely affecting its performance. When the Sn content reaches 4 at.%, the compressive strength has already decreased by 10 MPa compared to the alloy without Sn, and the fracture strain has decreased by 15%, resulting in poorer alloy performance. This study investigates the effects of Sn on the liquid phase sintering behavior and enhancing mechanical properties of TiAl alloys containing high-melting-point Nb. When the Sn content is below 2 at.%, Ti-18Al-28Nb-xSn alloy shows excellent density and mechanical properties. This study demonstrates the advantages of the low-melting-point alloying element Sn combining cold isostatic pressing and pressureless sintering in the preparation of the Ti-18Al-28Nb alloys, and it narrows the effective range of Sn content that optimizes the properties in TiAlNb alloys. This study provides valuable insights for research on the replacement of SPS and HIP in specific situations such as complex component formation and offers a reference for the development of densification sintering of alloys with high-melting-point elements assisted by low-melting-point alloys.

## Figures and Tables

**Table 1:** Chemical compositions (at.%) of various samples.

Element Content (at.%)				
Alloy	Ti	Al	Nb	Sn
S1	54	18	28	0
S2	53.5	18	28	0.5
S3	53	18	28	1
S4	52	18	28	2
S5	50	18	28	4

**Table 2:** The atomic percentage of each element in the α_2_ phase.

Element Content (at.%)				
Alloy (α_2_)	Ti	Al	Nb	Sn
S2	61.32	16.94	13.29	8.45
S3	62.56	22.11	12.99	2.34
S4	63.09	20.87	12.28	3.77
S5	61.10	17.66	13.70	7.54

**Table 3:** The atomic percentage of each element in the O phase.

Element Content (at.%)				
Alloy (O)	Ti	Al	Nb	Sn
S2	49.73	17.73	28.83	3.71
S3	54.98	18.82	24.83	1.37
S4	53.86	18.87	25.01	2.26
S5	49.12	19.02	28.29	3.57

## Data Availability

The original contributions presented in the study are included in the article, further inquiries can be directed to the corresponding author.
